# Application of Janus Kinase Inhibitors in Atopic Dermatitis: An Updated Systematic Review and Meta-Analysis of Clinical Trials

**DOI:** 10.3390/jpm11040279

**Published:** 2021-04-07

**Authors:** Hou-Ren Tsai, Jing-Wun Lu, Li-Yu Chen, Tai-Li Chen

**Affiliations:** 1 Department of Medical Education, Medical Administration Office, Hualien Tzu Chi Hospital, Buddhist Tzu Chi Medical Foundation, Hualien 970, Taiwan; melotsai0830@gmail.com; 2 Department of Physical Medicine and Rehabilitation, Hualien Tzu Chi Hospital, Buddhist Tzu Chi Medical Foundation, Hualien 970, Taiwan; jingwunlu@gmail.com; 3 Library of Hualien Tzu Chi Hospital, Buddhist Tzu Chi Medical Foundation, Hualien 970, Taiwan; rani37jason@gmail.com; 4 School of Medicine, Tzu Chi University, Hualien 970, Taiwan

**Keywords:** atopic dermatitis, eczema, JAK inhibitors, systematic review, meta-analysis, evidence-based medicine, immune-mediated skin diseases, target therapy, skin conditions and systemic inflammatory diseases

## Abstract

Janus kinase (JAK) inhibitors are promising treatments for atopic dermatitis (AD). The aim of this study was to assess the efficacy and safety of JAK inhibitors for AD treatment via the “Grading of Recommendations Assessment, Development, and Evaluation” approach. We identified 15 randomized controlled trials comparing oral or topical JAK inhibitors against placebo to treat AD. A random-effects meta-analysis was performed, and the numbers-needed-to-treat (NNTs)/numbers-needed-to-harm (NNHs) were calculated. Patients treated with JAK inhibitors were associated with higher rates of achieving eczema area and severity index-75 (rate ratio (RR): 2.84; 95% confidence interval (CI): 2.20–3.67; I^2^: 38.9%; NNT = 3.97), Investigator’s Global Assessment response (RR: 2.99; 95% CI: 2.26–3.95; I^2^: 0%; NNT = 5.72), and pruritus numerical rating scale response (RR: 2.52; 95% CI: 1.90–3.35; I^2^: 39.4%; NNT = 4.91) than those treated with placebo. Moreover, patients treated with JAK inhibitors had a higher risk of treatment-emergent adverse events (RR: 1.14; 95% CI: 1.02–1.28; I^2^: 52%; NNH = 14.80) but not adverse events leading to drug discontinuation. According to the evidence-based results, JAK inhibitors are potentially effective strategies (certainty of evidence: “moderate”) for treating AD with tolerable side effects (certainty of evidence: “low”). Nevertheless, long-term follow-up is required.

## 1. Introduction

Atopic dermatitis (AD) is the most common inflammatory skin disease affecting approximately 20% of children and 10% of adults worldwide [1,2]. This chronic condition is characterized by intense pruritus and recurrent eczematous lesions and has a considerable negative impact on patients’ quality of life [3]. Moreover, AD patients may display different clinical patterns depending on their age, ethnicity, and underlying mechanisms [4]. There are three main types of AD patterns: the persistent form where AD appears in childhood and then persists into adulthood; the relapsing form, in which AD occurs in childhood and recurs in adulthood with a symptom-free interval; and the adult-onset AD, the most difficult type to detect, where the disease is firstly observed in adulthood [5]. Due to its various presentations, the diagnosis and treatment of AD remained a challenge for clinicians [6].

While topical interventions are the mainstay treatment of AD, systemic therapy is recommended for patients with inadequate treatment response [7,8,9]. Advances have been made in systemic AD therapy owing to an improved understanding of the molecular mechanism of AD [10]. Innovative treatment options are available, such as dupilumab (obstructing alpha subunit of the IL-4 receptors), crisaborole (blocking phosphodiesterase 4), and lebrikizumab (preventing IL-13Rα1/IL-4Rα heterodimerization) [11]. Of note, the upregulation of key cytokines (interleukin (IL)-4, IL-5, and IL-13) and subsequent activation of the Janus kinase/signal transducer and transcription (JAK/STAT) pathways play important roles in the complex mechanism of AD [12,13]. In a growing number of preclinical studies, the inhibition of intracellular JAK/STAT signal transmission has demonstrated therapeutic potential [14,15].

Since their first approval, JAK inhibitors have become a promising AD treatment option [16]. In randomized controlled trials (RCTs), several JAK inhibitors (oral/topical) have significantly improved the clinical outcomes of patients with inadequate responses to other therapies [17,18]. An integrated safety analysis indicated similar incidences of adverse events (AEs) between JAK inhibitor application and placebo [19]. An earlier meta-analysis showed that JAK inhibitors lowered the eczema area and severity index (EASI) and pruritus scores [20]. Nevertheless, the sample sizes of the enrolled studies were small, and no phase III RCTs were included [20]. Considering the rapid developments in this field of study, an updated literature review with a meta-analysis is warranted to improve statistical power and provide supporting evidence for current guidelines.

In the present study, we performed a systematic review and meta-analysis of RCTs to compile evidence on the application of JAK inhibitors in the management of AD. We also graded certainty of evidence (CoE) based on the “Grading of Recommendations Assessment, Development, and Evaluation” (GRADE) approach and calculated the numbers-needed-to-treat (NNTs) and numbers-needed-to-harm (NNHs) to facilitate decision-making in clinical practice.

## 2. Materials and Methods

This study was conducted in accordance with the recommendations of the Cochrane Handbook for Systematic Reviews of Interventions (v. 6.2) [21] and reported based on the Preferred Reporting Items for Systematic Reviews and Meta-Analyses (PRISMA) statement [22]. The methodology was prespecified and registered on the PROSPERO website (Registration No. CRD42020173098). Two reviewers (H.R. Tsai and J.W. Lu) independently searched for suitable records, extracted the data, and evaluated the quality of the included studies. In the event of any discrepancy, a third reviewer (T.L. Chen) provided consensus or contributed to the discussions.

### 2.1. Data Sources and Literature Search

Relevant publications indexed in electronic databases (MEDLINE, Embase, Cochrane Central Register of Controlled Trials, and Web of Science) between database inception and 1 February 2021 were searched. The keywords “atopic dermatitis” and “Janus kinase inhibitor” and their synonyms and derivatives were used for the search. Details of the search strategies are described in Table S1 in the Supplementary Materials. No language restriction was applied. To identify unpublished or ongoing trials, the trial registries of the US National Institutes of Health (https://clinicaltrials.gov/ accessed on 2 February 2021), International Clinical Trials Registry Platform (https://www.who.int/ictrp/en/ accessed on 2 February 2021), and European Union Drug Regulating Authorities Clinical Trials Database (https://eudract.ema.europa.eu/ accessed on 3 February 2021) were searched. The bibliographies of available review articles and meta-analyses were also examined to find additional candidate studies. The literature search was performed with the assistance of a librarian (L.Y. Chen) at Hualien Tzu Chi Hospital.

### 2.2. Study Selection and Eligibility

Only RCTs were enrolled to avoid potential selection and confounding bias [21,23]. Studies comparing oral/topical JAK inhibitors against placebo were included with no defined limitations on age, sex, ethnicity, AD severity, or treatment duration of participants. Case reports, letters, editorials, review articles, conference abstracts, and in vivo studies involving animals were excluded.

### 2.3. Data Extraction and Efficacy and Safety Outcomes

The extracted data comprised study information (first author, publication year, clinical trial identifier, study design, and study period), patient characteristics (sample size, age, definition criteria, and AD severity), details of the various JAK inhibitors and placebos used (administration route, dosage, frequency, mode of action, and endpoint), and outcomes (efficacy and safety). Potential conflicts of interest were also listed.

The efficacy outcomes included (1) a ≥ 75% decrease in EASI from baseline (EASI-75 response), (2) an Investigator’s Global Assessment (IGA) score of 0 (clear) or 1 (almost clear) with a ≥ 2-point reduction from baseline (IGA response), and (3) a ≥ 4-point decrease from baseline in pruritus numerical rating scale response (pruritus-NRS response). The safety outcomes included the development of treatment-emergent AEs (TEAEs) and AEs that lead to drug discontinuation.

### 2.4. Risk of Bias Assessment

The methodological quality of the RCTs was appraised using the Cochrane Risk of Bias Tool (RoB v. 2.0, The Cochrane Collaboration) [24].

### 2.5. Data Synthesis and Statistical Analyses

Pooled estimates and their confidence intervals (CIs) were obtained with a random-effects meta-analysis model (DerSimonian–Laird estimator) [21] based on the assumption of substantial clinical heterogeneity. Rate ratios or relative risks (RRs) and 95% CIs were used to evaluate the efficacy and safety outcomes. If the desired effect estimates were inadequate for data synthesis, the corresponding authors were contacted to obtain relevant information. Statistical significance was indicated by *p* < 0.05 and a CI not containing 1. All statistical analyses were conducted using Stata v.16 (StataCorp, College Station, TX, USA).

A pairwise meta-analysis comparing the effect of JAK inhibitors against placebos was conducted. However, when a multiple-arm RCT was enrolled, a unit-of-analysis error may arise if the same group of participants is included twice in the same meta-analysis (for example, if “dose 1 vs. placebo” and “dose 2 vs. placebo” are both included in the same analysis, with the same placebo patients in both comparisons) [25]. Therefore, when an enrolled RCT included more than two arms, the reported results were combined to create a single pairwise comparison against placebos [21]. This method had been utilized in previous studies [26,27].

Between-study heterogeneity was quantified using Cochran’s Q and I^2^ statistics [28]. *p* < 0.01 and I^2^ ≥ 50% indicated substantial heterogeneity. To identify the potential sources of heterogeneity, several subgroup analyses were conducted according to the administration route, AD severity, participant age, mode of action, and different JAK inhibitors at different time points. Meta-regression analyses were also performed to explore potential effect modifiers, which refer to the explanatory variables of the overall effect estimates that may contribute to the heterogeneity [21]. Visual inspection of the funnel plot and Egger’s regression test were used to assess publication bias [21].

Concerning the possibility of producing false-positive results when using the DerSimonian–Laird method, we applied the Hartung–Knapp–Sidik–Jonkman (HKSJ) method for sensitivity analyses [29]. This method widens the CIs to reflect uncertainty in estimating between-study heterogeneity, especially when the study number is less than 20 [21,30]. The HKSJ method had been widely used in previous meta-analyses [31,32,33].

The GRADE approach was adopted to summarize the CoE at the outcome level [34] and judged by all authors. In the event of disagreement, discussions were undertaken to arrive at a consensus for each outcome. CoE, classified as high, moderate, low, or very low, refers to the confidence that the true effect lies in a particular range [34]. The CoE was downgraded by one level if a serious flaw was present in the domains of risk of bias, inconsistency, indirectness, imprecision, and publication bias [34]. The NNT and NNH were calculated to evaluate the evidence-based efficacy and safety of JAK inhibitors in the management of AD [34].

## 3. Results

### 3.1. Search Results

A PRISMA flowchart of the process of study selection is shown in Figure S1. Initially, a total of 1237 records were retrieved. After screening the titles, abstracts, and full texts, 14 original articles involving 15 RCTs were eligible for a quantitative meta-analysis. These comprised seven phase III trials [19,35,36,37,38,39], seven phase II trials [40,41,42,43,44,45,46], and one phase I trial [47]. 

### 3.2. Characteristics of Eligible Studies

The demographic data and relevant outcomes are summarized in Table 1. We included 4367 patients with AD in this meta-analysis and assessed seven different JAK inhibitors. Four of them (abrocitinib, baricitinib, gusacitinib, and upadacitinib) were orally administered, while the remaining three (delgocitinib, ruxolitinib, and tofacitinib) were topically administered. All eligible studies had participants with a documented history of inadequate treatment response to topical corticosteroids/calcineurin inhibitors. Guttman-Yassky et al. also recruited participants with a documented history of inadequate treatment response to systemic corticosteroids or immunosuppressants [42]. Most eligible studies did not report participants with failure of systemic treatment but a washout period for systemic steroid, immunosuppressive, and biologic treatments.

Most of the enrolled trials involved adult patients with moderate to severe AD. Three studies involved children or adolescents, while another three included patients with mild to moderate AD. All enrolled studies had declared their conflict of interest with an institution or a company.

### 3.3. Risk of Bias Assessment

We used the Risk-of-Bias VISualization tool to create “traffic light” plots of domain-level judgments [48]. Most of the enrolled RCTs were judged to have a “low” risk of bias (Figure S2).

### 3.4. Efficacy Outcomes

#### 3.4.1. EASI-75 Response

**Figure 1:**
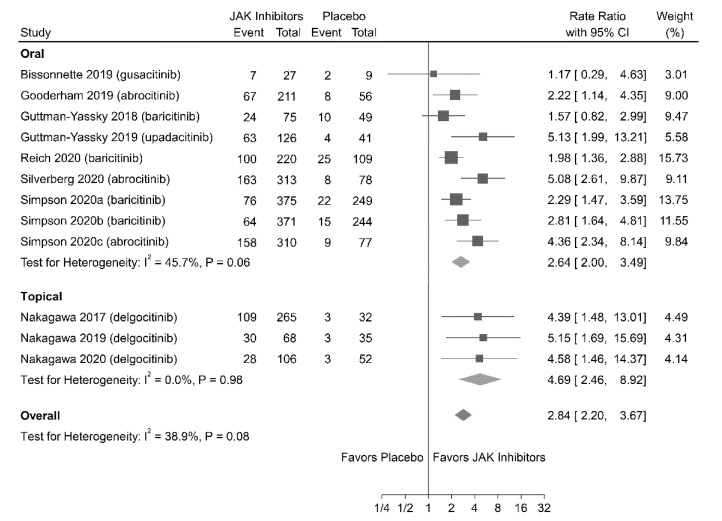
Association of Janus kinase (JAK) inhibitors versus placebo achieving at least a 75% improvement in Eczema Area and Severity Index (EASI) score from the baseline (EASI-75 response). CI, confidence interval.

Twelve studies (involving 3498 patients) reported EASI-75 response as the indicator of efficacy outcome. A random-effects meta-analysis showed that patients treated with JAK inhibitors (both oral and topical) were associated with higher rates of achieving EASI-75 response (RR = 2.84; 95% CI = 2.20–3.67) than those treated with placebo (Figure 1); no significant heterogeneity was observed (I^2^ = 38.9%; *P* = 0.08).

#### 3.4.2. IGA Response

**Figure 2:**
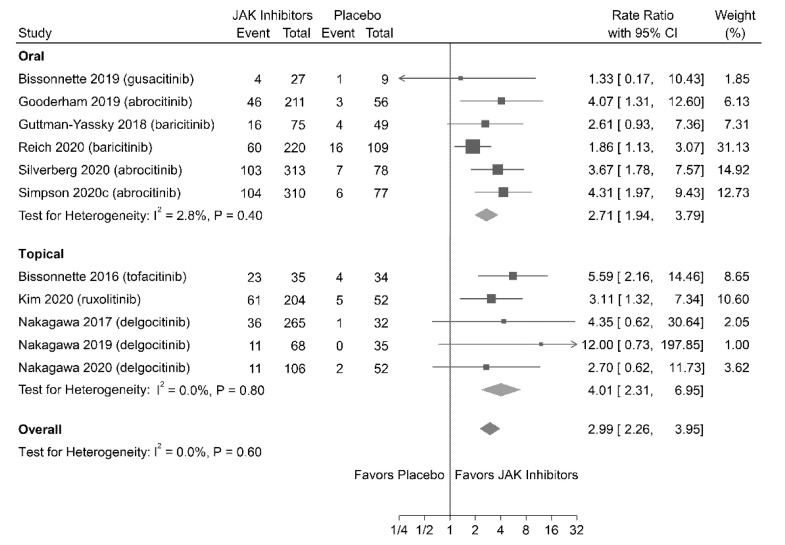
Association of Janus kinase (JAK) inhibitors versus placebo achieving of an Investigator Global Assessment (IGA) of 0 (clear) or 1 (almost clear) with at least a two-point reduction from the baseline (IGA response). CI, confidence interval.

Eleven studies (involving 2417 patients) reported IGA responses. Figure 2 shows that patients treated with JAK inhibitors (both oral and topical) were associated with higher rates of achieving IGA responses (RR = 2.99; 95% CI = 2.26–3.95) than those treated with placebo; no significant heterogeneity was observed (I^2^ = 0%; *P* = 0.60).

#### 3.4.3. Pruritus-NRS Response

**Figure 3:**
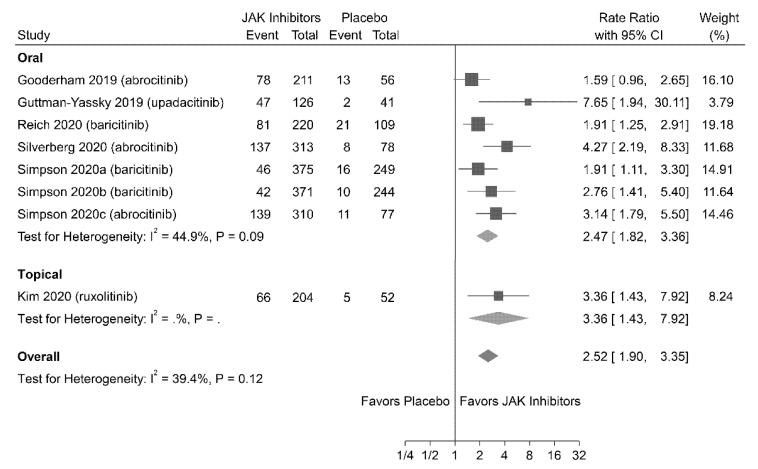
Association of Janus kinase (JAK) inhibitors versus placebo achieving at least a four-point improvement in pruritus Numerical Rating Scale (NRS) score from the baseline (pruritus-NRS response). CI, confidence interval.

Eight studies (involving 3036 patients) reported pruritus-NRS responses. The meta-analysis revealed that patients treated with JAK inhibitors were associated with higher rates of achieving pruritus-NRS responses (RR = 2.52; 95% CI = 1.90–3.35) compared with those treated with placebo (Figure 3). No significant heterogeneity was observed (I^2^ = 39.4%; *P* = 0.12).

#### 3.4.4. Subgroup Analyses and Meta-Regression of Efficacy Outcomes

The subgroup analyses indicated that JAK inhibitors improved AD. From the meta-regression analyses, we identified age as a potential effect modifier of EASI-75 responses. Studies involving children and adolescents showed a higher RR of achieving an EASI-75 response than those exclusively involving adults (Table S2). According to the subgroup analyses of different JAK inhibitors at different time points, gusacitinib was unlikely to achieve EASI-75 and IGA responses (Table S3).

### 3.5. Safety Outcomes

#### 3.5.1. TEAEs

Twelve studies (involving 3402 patients) were included in the analysis of TEAEs. AD patients treated with JAK inhibitors had relatively higher risks of developing TEAEs (RR = 1.14; 95% CI = 1.02–1.28) than those treated with placebo (Table 2). Most TEAEs were tolerable; nasopharyngitis was the most reported event (Table 3), followed by upper respiratory tract infection, headache, nausea, diarrhea, elevated blood creatine phosphokinase levels, and acne. Herpesvirus infection was reported in patients treated with abrocitinib or baricitinib. However, substantial heterogeneity was observed among studies (I^2^ = 52%; *P* = 0.023). Table 2 shows several potential effect modifiers accounting for the considerable heterogeneity among the TEAEs, including administration route, AD severity, and treatment duration. Patients who were administered oral JAK inhibitors experienced moderate to severe AD at baseline. Those treated with JAK inhibitors for >12 weeks were more likely to develop TEAEs.

#### 3.5.2. AEs Leading to Drug Discontinuation

Fourteen studies (involving 3926 patients) were included in the analysis of AEs that led to drug discontinuation. Table 2 shows that patients who were administered with JAK inhibitors were unlikely to have higher risks of developing AEs (RR = 0.89; 95% CI = 0.57–1.38) than those treated with placebo. No significant heterogeneity was detected (I^2^ = 0%; *P* = 0.62).

#### 3.5.3. Sensitivity Analyses

As shown in Table S4, a sensitivity analysis of the overall effects on each outcome before and after the application of modified HKSJ adjustment yielded similar results, demonstrating the robustness of the findings.

### 3.6. Publication Bias

No publication bias was detected after the visual inspection of the funnel plot or Egger’s regression test except for the pruritus-NRS response outcome (Figure S3).

### 3.7. GRADE Approach for CoE

We presented the CoE using a modified GRADE evidence profile (Table 4) on the GRADEpro website (https://gdt.gradepro.org/ accessed on 23 February 2021). The GRADE guidelines [49] indicated that publication bias levels were downgraded for studies that identified conflicts of interest. The overall CoE of the efficacy outcomes was deemed “moderate.” Owing to the “high” bias risk of BREEZE-AD4 2020, the overall CoE of the safety outcomes was considered “low.” The NNTs for patients achieving IGA, EASI-75, and pruritus-NRS response were 3.97, 5.72, and 4.91, respectively. The NNH for patients experiencing TEAEs was 14.80.

## 4. Discussion

Our study provides evidence that JAK inhibitors are more effective in achieving EASI-75, IGA, and pruritus-NRS responses than placebo in patients with AD. AD patients treated with JAK inhibitors had relatively higher risks of developing TEAEs, but no higher risks were observed regarding AEs leading to drug discontinuation. From the viewpoint of evidence-based medicine based on the GRADE approach, the overall CoE of the efficacy outcomes was “moderate,” while that of the safety outcomes was considered “low”.

A previous meta-analysis of five RCTs demonstrated that JAK inhibitors lowered EASI and pruritus scores [20], which is consistent with the findings of this study. However, in contrast to the former study with a small sample size that represented only four countries, we included 15 multi-center RCTs in more than 10 countries and performed subgroup analyses and meta-regressions to identify potential effect modifiers of the efficacy and safety outcomes. Additionally, sensitivity analyses confirmed the robustness of our results. We also applied a GRADE assessment and furnished the NNT and NNH results to facilitate evidence-based decisions in the treatment of AD in clinical practice. Hence, our updated meta-analysis is robust and provides more evidence than a previously published meta-analysis [20].

Given that different kinds of JAK inhibitors seemed to have different effects in AD patients, there were no head-to-head comparisons between JAK inhibitors. According to our subgroup analyses of different JAK inhibitors at different time points, gusacitinib was unlikely to achieve EASI-75 and IGA responses. In the original article, gusacitinib showed benefits in achieving EASI-50 but not EASI-75 [47]. This may also be subject to the lack of statistical power due to an insufficient number of participants. Additionally, topical delgocitinib had higher rates of achieving EASI-75 response than placebo but not IGA response. Other topical agents such as tofacitinib or ruxolitinib demonstrated better response than placebos regarding IGA and pruritus-NRS responses. As seen in Table 3, ruxolitinib and delgocitinib seemed to have fewer TEAEs than other JAK inhibitors. This discovery is important to clinicians because these topical agents may serve as better options than oral forms for their effectiveness and fewer side effects.

We selected EASI as an efficacy outcome because it is adequately validated and recommended for the evaluation of the clinical signs of AD in RCTs [50,51]. However, an EASI assessment is not always feasible in routine practice as it is complex and time-consuming [50,51]. IGA is a rapid and easily interpreted alternative to EASI, and it should be included in the measurement outcomes for clinical trial approval under US drug regulations [52]. Despite the lack of standardization and validation, our meta-analysis of the IGA responses indicated less heterogeneity. Moreover, because EASI and IGA were evaluated by medical professionals, a patient-oriented scale is also critical for the assessment of drug efficacy. Consequently, considering chronic and intense pruritus is the major symptom observed in AD patients, we analyzed the previously validated pruritus-NRS as an efficacy outcome [53].

We performed several subgroup analyses and observed that children and adolescents had a higher RR of attaining an EASI-75 response. Only one RCT exclusively assessed pediatric patients. Therefore, no conclusions could be drawn regarding the efficacy of JAK inhibitors in this population.

The administration of JAK inhibitors was associated with an elevated risk of TEAEs. Nevertheless, most TEAEs were mild and tolerable. Nasopharyngitis, headache, and upper respiratory tract infection were the most common TEAEs observed in the enrolled RCTs, consistent with the findings of other systemic immunomodulators in AD management [54,55]. Recently, the warning black box issued by the Food and Drug Administration (FDA) reported an increased risk of serious cardiovascular problems with an oral form of tofacitinib, a JAK inhibitor in treating rheumatoid arthritis (RA) and ulcerative colitis [56]. Among the management of RA patients, JAK inhibitors (baricitinib, filgotinib, and tofacitinib) have also received warnings from the FDA about the increased risk of thromboembolic events and the higher rates of all-cause mortality. Even though the pharmacological features of the topical tofacitinib in AD may differ from that of oral usage in the above circumstances, these safety issues should not be ignored.

Recent studies have reported that AD exhibits complex dysregulations and multiple clinical phenotypes [57,58,59]. By contrast, between-study heterogeneity with seven distinct JAK inhibitors was observed to be unremarkable in most analyses. We hypothesized that the intracellular blockade by JAK inhibitors results in relatively less interference with the extracellular environment [60,61] and is indicated by the marked homogeneity among various types of JAK inhibitors in clinical settings. Furthermore, we identified administration route, severity, and treatment duration as potential effect modifiers for TEAE outcomes (Table 2). Systemic drug absorption via oral administration, severe inflammatory reactions in patients with moderate to severe AD, and longer treatment durations over 12 weeks could explain the results we obtained from the meta-regression analyses.

NNT/NNH is the average number of patients undergoing treatment with a particular therapy to achieve one additional positive/negative outcome compared with the placebo [62]. NNT < 10 and NNH ≥ 10 indicate “clinically desirable” benefit or harm of a particular therapeutic intervention [63]. In this study, the NNT was <10 for all efficacy outcomes, representing desirable effects compared with placebos. Despite the NNH for TEAEs being 14.80, the AEs were relatively innocuous. We believe that our findings could be helpful for clinical dermatologists in treating patients with AD.

A key strength of our study is the updated literature review and meta-analysis via an evidence-based approach. We provide CoE based on the GRADE system and calculate the NNTs/NNHs, which could guide clinicians in decision-making for AD treatment. However, the findings of this study must be considered with certain limitations in mind. First, we only compared the effects of JAK inhibitors against placebos. Comparisons of different types of JAK inhibitors were not performed in this study. Accordingly, we assume rigorous head-to-head RCTs to be beneficial in the comparison of the efficacy and safety outcomes of various JAK inhibitors. Second, we could not draw a firm conclusion concerning the efficacy of JAK inhibitors for the treatment of pediatric patients with AD because only one RCT that assessed patients aged less than 18 years was enrolled in this study. Given that pediatric AD is common, additional trials to clarify the effectiveness and safe dosage of JAK inhibitors in children and adolescents with AD are required. Third, it has only been five years since JAK inhibitors were approved for the treatment of AD; consequently, only a few RCTs with long-term follow-ups are available at present. Because we could only elucidate the short-term effects of JAK inhibitors, the results of this meta-analysis do not guarantee the long-term safety of JAK inhibitors. Hence, the evidence-based results presented herein must be interpreted with caution.

## 5. Conclusions

This systematic review and meta-analysis provide updated evidence for current AD guidelines. The findings demonstrate that JAK inhibitors have favorable efficacy (overall CoE: “moderate”) in the treatment of AD with tolerable safety issues (overall CoE: “low”). However, planned prospective studies involving long-term follow-up of AEs and cost-effective analyses could aid clinical decisions in the application of JAK inhibitors for the treatment of AD.

## Figures and Tables

**Table 1:** Characteristics of studies included in the meta-analysis.

Source	Clinical Trial Identifier	Study Design	Study Period	No. of Participants (Age)	Definition of AD	Severity of AD	Interventions	Mechanism of Inhibition	Endpoint	Efficacy Outcomes	Safety Outcomes	COI
Bissonnette 2016	NCT02001181	Phase II RCT	December 2013–September 2014	69 (18–60 y)	Hanifin and Rajka criteria	Mild to moderate	Treatment: topical tofacitinib, 2% twice daily Placebo: topical control vehicle twice daily	JAK1 and JAK3	Week 4	IGA, EASI, and BSA	TEAEs and SAEs	Yes
Bissonnette 2019	NCT03139981	Phase I RCT	April 2017– November 2017	36 (18–75 y)	AAD guideline	Moderate to severe	Treatment: oral gusacitinib, 20 mg, 40 mg, 80 mg once daily Placebo: oral vehicle once daily	JAK1, JAK2, JAK3, TYK2, and SYK	Week 4	IGA, EASI, pruritus NRS, and BSA	TEAEs, SAEs, and AEDC	Yes
Gooderham 2019	NCT02780167	Phase II RCT	April 2016– April 2017	267 (18–75 y)	AAD guideline	Moderate to severe	Treatment: oral abrocitinib, 10 mg, 30 mg, 100 mg, 200 mg once daily Placebo: oral control vehicle once daily	JAK1	Week 12	IGA, EASI, pruritus NRS, BSA, SCORAD, DLQI, HADS, and POEM	TEAEs and SAEs	Yes
Guttman-Yassky 2018	NCT02576938	Phase II RCT	February 2016– March 2017	124 (≥18 y)	Hanifin and Rajka criteria	Moderate to severe	Treatment: oral baricitinib, 2 mg and 4 mg once daily plus TCSPlacebo: oral control vehicle once daily	JAK1 and JAK2	Week 16	IGA, EASI, pruritus NRS, SCORAD, DLQI, and POEM	TEAEs, SAEs, and AEDC	Yes
Guttman-Yassky 2019	NCT02925117	Phase II RCT	November 2016– April 2017	167 (18–75 y)	Hanifin and Rajka criteria	Moderate to severe	Treatment: oral upadacitinib, 7.5 mg, 15 mg, 30 mg once daily Placebo: oral control vehicle once daily	JAK1	Week 16	IGA, EASI, pruritus NRS, BSA, SCORAD, and POEM	TEAEs, SAEs, and AEDC	Yes
Kim 2020	NCT03011892	Phase II RCT	January 2017– November 2017	307 (18–70 y)	NA	Mild to moderate	Treatment: topical ruxolitinib, 0.15%, 0.5%, 1.5% once daily, and 1.5% twice daily; Placebo: topical control vehicle twice daily	JAK1 and JAK2	Week 8	pruritus NRS, and Skindex-16	TEAEs, SAEs, and AEDC	Yes
Nakagawa 2017	JapicCTI-152887	Phase II RCT	April 2015– May 2016	327 (16–65 y)	JDA guideline	Moderate to severe	Treatment: topical delgocitinib, 0.25%, 0.5%, 1%, 3% twice daily Placebo: topical control vehicle twice daily	JAK1, JAK2, JAK3, and TYK2	Week 4	IGA, mEASI, pruritus NRS, and BSA	SAEs and AEDC	Yes
Nakagawa 2019	JapicCTI-173553	Phase II RCT	March 2017– February 2018	103 (2–15 y)	JDA guideline	Mild to moderate	Treatment: topical delgocitinib, 0.25%, 0.5% twice daily Placebo: topical control vehicle twice daily	JAK1, JAK2, JAK3, and TYK2	Week 4	IGA, mEASI, pruritus NRS, and BSA	SAEs and AEDC	Yes
Nakagawa 2020	JapicCTI-173554	Phase III RCT	March 2017– September 2018	158 (≥16 y)	JDA guideline	Moderate to severe	Treatment: topical delgocitinib, 0.5% twice daily Placebo: topical control vehicle twice daily	JAK1, JAK2, JAK3, and TYK2	Week 4	IGA, mEASI, pruritus NRS, BSA, and Skindex-16	TEAEs, SAEs, and AEDC	Yes
Reich 2020	NCT03733301 (BREEZE-AD7)	Phase III RCT	November 2018–August 2019	329 (≥18 y)	AAD guideline	Moderate to severe	Treatment: oral baricitinib, 2 mg, 4 mg once daily plus TCS Placebo: oral control vehicle once daily	JAK1 and JAK2	Week 16	IGA, EASI-50, EASI-75, EASI-90, pruritus NRS, pain NRS, SCORAD, ADSS, POEM, HADS, DLQI, and WPAI	TEAEs, SAEs, and AEDC	Yes
Silverberg 2020	NCT03575871 (JADE MONO-2)	Phase III RCT	June 2018– August 2019	391 (≥12 y)	Hanifin and Rajka criteria	Moderate to severe	Treatment: oral abrocitinib, 100 mg and 200 mg once daily Placebo: oral control vehicle once daily	JAK1	Week 12	IGA, EASI, pruritus NRS, PSAAD, DLQI, CDLQI, POEM, and HADS	SAEs and AEDC	Yes
Simpson 2020a	NCT03334396 (BREEZE-AD1)	Phase III RCT	November 2017–January 2019	624 (≥18 y)	AAD guideline	Moderate to severe	Treatment: oral baricitinib, 1 mg, 2 mg, 4 mg once daily Placebo: oral control vehicle once daily	JAK1 and JAK2	Week 16	IGA, EASI, pruritus NRS, pain NRS, SCORAD, and ADSS	TEAEs, SAEs, and AEDC	Yes
Simpson 2020b	NCT03334422 (BREEZE-AD2)	Phase III RCT	November 2017–December 2018	615 (≥18 y)	AAD guideline	Moderate to severe	Treatment: oral baricitinib, 1 mg, 2 mg, 4 mg once daily Placebo: oral control vehicle once daily	JAK1 and JAK2	Week 16	IGA, EASI, pruritus NRS, pain NRS, SCORAD, and ADSS	TEAEs, SAEs, and AEDC	Yes
Simpson 2020c	NCT03349060 (JADE MONO-1)	Phase III RCT	December 2017–March 2019	387 (≥12 y)	Hanifin and Rajka criteria	Moderate to severe	Treatment: oral abrocitinib, 100 mg and 200 mg once daily Placebo: oral control vehicle once daily	JAK1	Week 12	IGA, EASI, pruritus NRS, PSAAD, DLQI, CDLQI, and POEM	TEAEs, SAEs, and AEDC	Yes
BREEZE-AD4 2020 *	NCT03428100 (BREEZE-AD4)	Phase III RCT	May 2018	463 (≥18 y)	AAD guideline	Moderate to severe	Treatment: oral baricitinib, 1 mg, 2 mg, 4 mg once daily Placebo: oral control vehicle once daily	JAK1 and JAK2	Week 16	NA	TEAEs, SAEs, and AEDC	Yes

AAD, American Academy of Dermatology; AD, atopic dermatitis; ADSS, atopic dermatitis sleep scale; AE, adverse event; AEDC, adverse events leading to drug discontinuation; BSA, body surface area; CDLQI, children’s dermatology life quality index; COI, conflict of interest; DLQI, dermatology life quality index; EASI, eczema area and severity index; HADS, hospital anxiety and depression scale; IGA, investigator global assessment; JAK, Janus kinase; JDA, Japanese Dermatological Association; mEASI, modified eczema area and severity index; NA, not applicable; NRS, numerical rating scale; POEM, patient-oriented eczema measure; PSAAD, pruritus and symptoms assessment for atopic dermatitis; RCT, randomized controlled trial; SAE, serious adverse event; SCORAD, scoring atopic dermatitis; SYK, spleen tyrosine kinase; TEAE, treatment-emergent adverse event; TYK: tyrosine kinase; WPAI, work productivity and activity impairment questionnaire * BREEZE-AD4 is an ongoing RCT. Its safety outcomes were presented in Bieber et al. (2020).

**Table 2:** Safety outcomes of Janus kinase inhibitors.

	Treatment-Emergent Adverse Events				Meta-Regression		Adverse Events Leading to Drug Discontinuation				Meta-Regression	
Subgroups	No. of Studies	Pooled RR (95% CI)	*p*-Value	I^2^ (%)	τ^2^	*p*-Value	No. of Studies	Pooled RR (95% CI)	*p*-Value	I^2^ (%)	τ^2^	*p*-Value
Overall	12	1.14 (1.02 to 1.28) *	0.023	52.0			14	0.89 (0.57 to 1.38)	0.621	0.0		
Route of administration					0.013	0.033					0	0.064
Oral	9	1.18 (1.06 to 1.32) **	0.003	48.3			9	1.03 (0.64 to 1.64)	0.917	0.0		
Topical	3	0.77 (0.49 to 1.20)	0.255	25.2			5	0.26 (0.07 to 1.02)	0.054	0.0		
Severity of atopic dermatitis					0.012	0.021					0	0.036
Mild to moderate	2	0.73 (0.47 to 1.13)	0.163	33.1			3	0.16 (0.03 to 0.84) *	0.031	0.0		
Moderate to severe	10	1.18 (1.06 to 1.31) **	0.002	43.7			11	1.01 (0.64 to 1.60)	0.929	0.0		
Age of participants					0.019	0.483					0	0.200
Adults only	10	1.12 (0.99 to 1.26)	0.068	55.3			9	1.10 (0.63 to 1.93)	0.728	0.0		
Contain children or adolescents	2	1.41 (1.06 to 1.88) *	0.019	0.0			5	0.60 (0.29 to 1.26)	0.193	0.0		
Mechanism of action					0.012	0.062					0	0.750
Selective for JAK1 inhibition	3	1.29 (1.11 to 1.50) *	0.001	0.0			3	0.77 (0.39 to 1.52)	0.456	0.0		
Selective for JAK1/JAK2 inhibition	6	1.14 (0.98 to 1.31)	0.082	59.4			6	1.22 (0.60 to 2.51)	0.582	13.3		
Selective for JAK1/JAK3 inhibition	1	0.56 (0.32 to 1.00) *	0.049	NA			1	0.14 (0.01 to 2.59)	0.186	NA		
Pan-JAK inhibition	2	0.99 (0.66 to 1.48)	0.917	0.0			4	0.55 (0.11 to 2.63)	0.504	0.0		
Treatment duration					0.013	0.026					0	0.134
<12 weeks	4	0.84 (0.63 to 1.11)	0.223	8.2			6	0.37 (0.11 to 1.26)	0.120	0.0		
≥12 weeks	8	1.20 (1.07 to 1.34) **	0.002	52.5			8	1.01 (0.63 to 1.63)	0.965	0.0		

* *p* < 0.05; ** *p* < 0.01; CI, confidence interval; RR, risk ratio.

**Table 3:** Treatment-emergent adverse events reported in the studies included in the meta-analysis.

JAK Inhibitors by Mechanism	No. of Patients	No. (%) of Patients with Common TEAEs							
		Nasopharyngitis	URTI	Headache	Nausea	Diarrhea	Blood CPK Increase	Acne	Herpes Viral Infection
Selective for JAK1 inhibition									
Abrocitinib	834	73 (8.8)	85 (10.2)	64 (7.7)	94 (11.3)	10 (1.2)	8 (1.0)	11 (1.3)	10 (1.2)
Upadacitinib	126	9 (7.1)	17 (13.5)	10 (7.9)	7 (5.6)	4 (3.2)	7 (5.6)	12 (9.5)	0
Selective for JAK1/JAK2 inhibition									
Baricitinib	1318	118 (9.0)	36 (2.7)	64 (4.9)	2 (0.2)	25 (1.9)	27 (2.0)	5 (0.4)	74 (5.6)
Ruxolitinib	204	10 (4.9)	5 (2.5)	4 (2.0)	0	0	0	0	0
Selective for JAK1/JAK3 inhibition									
Tofacitinib	35	2 (5.7)	1 (2.9)	1 (2.9)	1 (2.9)	0	0	0	0
Pan-JAK inhibition									
Gusacitinib	27	3 (11.1)	0	7 (25.9)	5 (18.5)	3 (11.1)	0	0	0
Delgocitinib	439	28 (6.4)	0	0	0	0	0	4 (0.9)	0

AD, atopic dermatitis; AE, adverse event; NA, not applicable; RR, relative risk; SAE, serious adverse event; TEAE, treatment-emergent adverse event; URTI, upper respiratory tract infection.

**Table 4:** Certainty of evidence based on GRADE (Janus kinase inhibitors vs. placebo in atopic dermatitis).

Certainty Assessment							Summary of Findings	
Participants (Studies) Follow Up	Risk of Bias	Inconsistency	Indirectness	Imprecision	Publication Bias	Overall Certainty of Evidence	Relative Effect (95% CI)	NNTs or NNHs
EASI-75 response								
3498 (12 RCTs)	Not serious	Not serious	Not serious	Not serious	Likely ^a^	⨁⨁⨁◯ MODERATE	2.84 (2.20 to 3.67)	3.97
IGA response								
2417 (11 RCTs)	Not serious	Not serious	Not serious	Not serious	Likely ^a^	⨁⨁⨁◯ MODERATE	2.99 (2.26 to 3.95)	5.72
Pruritus-NRS response								
3036 (8 RCTs)	Not serious	Not serious	Not serious	Not serious	Likely ^a^	⨁⨁⨁◯ MODERATE	2.52 (1.90 to 3.35)	4.91
TEAEs								
3402 (12 RCTs)	Serious ^b^	Not serious	Not serious	Not serious	Likely ^a^	⨁⨁◯◯ LOW	1.14 (1.02 to 1.28)	14.80
AEs leading to drug discontinuation								
3926 (14 RCTs)	Serious ^b^	Not serious	Not serious	Not serious	Likely ^a^	⨁⨁◯◯ LOW	0.89 (0.57 to 1.38)	NR

AEs, Adverse Events; CI, confidence interval; GRADE, Grading of Recommendations Assessment, Development and Evaluation; IGA, Investigator’s Global Assessment; NNTs/NNHs, number-needed-to-treats/number-needed-to-harms; NR: not reasonable (no statistical significance in meta-analysis; therefore, calculation of this value is not reasonable); NRS, Numerical Rating Scale; RCT, randomized controlled trial; TEAEs, treatment emergent AEs. ^a^ Potential conflict of interest was indicated. ^b^ One study was judged as “high” bias risk.

## Data Availability

The data in this study were collated from published clinical trials, which could be accessed by the public.

## Supplementary Materials

The following are available online at https://www.mdpi.com/article/10.3390/jpm11040279/s1, Figure S1: Flowchart of the Material and Methods: PRISMA flow diagram of the study. RCT, randomized controlled trial, Figure S2: Summary of Risk of Bias Assessment, Figure S3: Funnel Plot of Pruritus-NRS Response. Table S1: Search strategies modified in MEDLINE (a), Embase (b), Cochrane CENTRAL (c), and Web of Science (d), Table S2: Subgroup analyses and meta-regressions of efficacy outcomes, Table S3: Subgroup analyses of efficacy outcomes for various Janus kinase inhibitors at different time points, Table S4: Sensitivity analysis of overall effects of each outcome before and after modified Hartung–Knapp–Sidik–Jonkman (HKSJ) adjustment.
