# Supplementary material for: Application of Janus Kinase Inhibitors in Atopic Dermatitis: An Updated Systematic Review and Meta-Analysis of Clinical Trials
Source: J Pers Med. 2021 Apr 7;11(4):279. doi: 10.3390/jpm11040279 (PMC8067719; doi:10.3390/jpm11040279)

Supplementary Materials

Figure S1. Flowchart of the Material and Methods: PRISMA flow diagram of the study. RCT, randomized controlled trial.

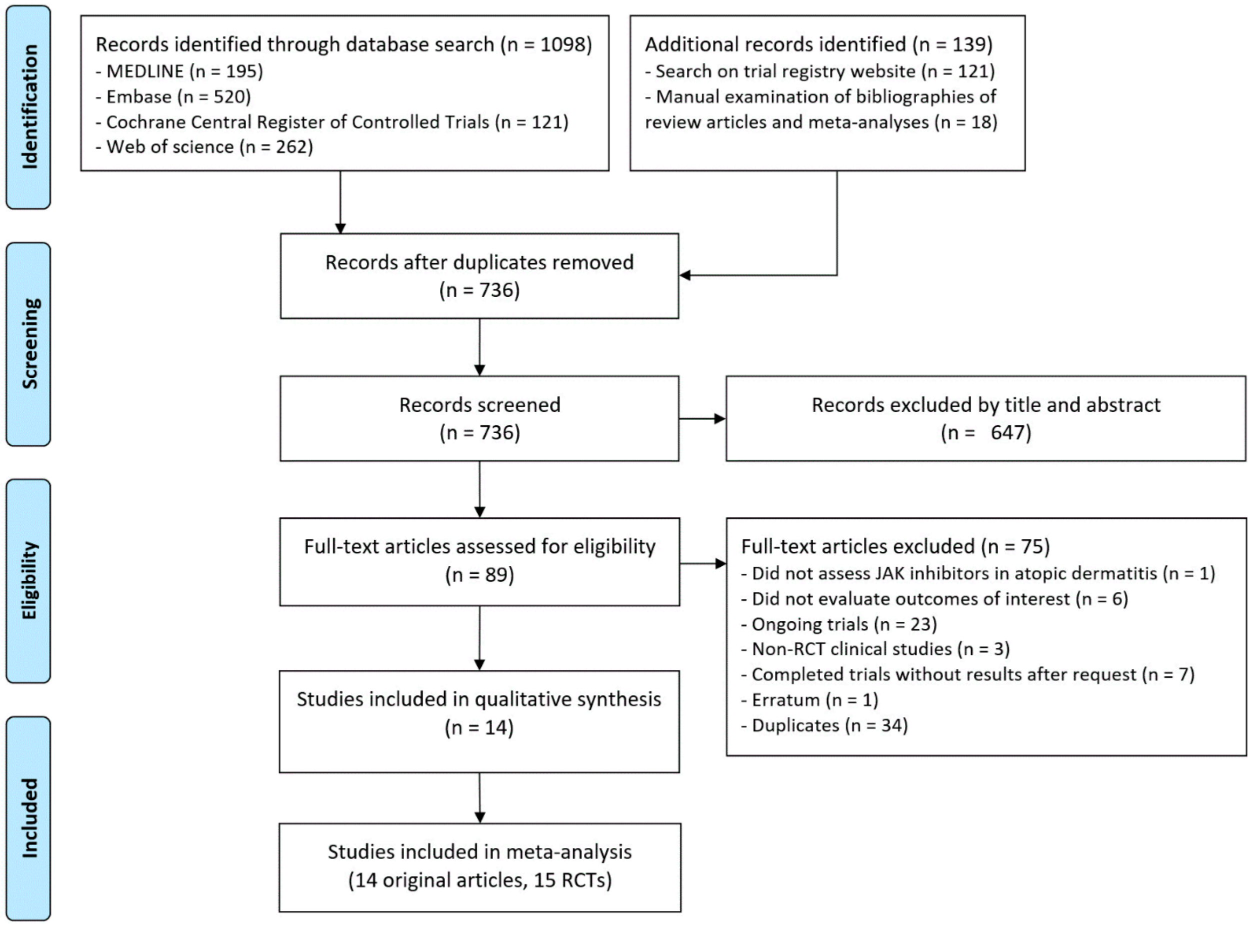

Table S1. Search strategies modified in MEDLINE (a), Embase (b), Cochrane CENTRAL (c), and Web of Science (d)

a. Search strategy in MEDLINE (via Ovid MEDLINE(R), 1946–present; search date: 2021/01/29)

#	Search syntax	Citations found
1	("atopic dermatitis" OR eczema*).mp	
2	exp "Dermatitis, Atopic"/ OR exp "Eczema"/	
3	((Janus ADJ3 kinase* ADJ3 inhibit*) OR (JAK* ADJ3 inhibit*) OR Abrocitinib OR "PF-04965842" OR Baricitinib OR LY3009104 OR Olumiant OR INCB028050 OR Delgocitinib OR "JTE-052" OR Gusacitinib OR ASN002 OR Ruxolitinib OR INCB018424 OR INCA24 OR Tofacitinib OR Tasocitinib OR "Tofacitinib citrate" OR Xeljanz OR CP690550 OR Upadacitinib OR "ABT-494" OR RINVOQ OR Cerdulatinib OR "RVT-502" OR PRT062070 OR Peficitinib OR ASP015K OR Filgotinib OR GLPG0634 OR Solcitinib OR "1206163-45-2" OR GLPG0778 OR GSK2586184 OR SHR0302 OR "ATI-502" OR "ATI 50002" OR "A 301" OR "PF-06700841" OR "RVT-501" OR Decernotinib OR "VX-509" OR Pacritinib OR SB1518 OR Oclacitinib OR Apoquel OR "PF-03394197" OR Fedratinib OR TG101348 OR SAR302503 OR "Fedratinib hydrochloride" OR Inrebic).mp	
4	exp "Janus Kinase Inhibitors"/	
5	(1 OR 2) AND (3 OR 4)	195

b. Search strategy in Embase (via Ovid, 1974–present; search date: 2021/01/29)

#	Search syntax	Citations found
1	("atopic dermatitis" OR eczema*).mp	
2	exp "atopic dermatitis"/ OR exp "eczema"/	
3	((Janus ADJ3 kinase* ADJ3 inhibit*) OR (JAK* ADJ3 inhibit*) OR Abrocitinib OR "PF-04965842" OR Baricitinib OR LY3009104 OR Olumiant OR INCB028050 OR Delgocitinib OR "JTE-052" OR Gusacitinib OR ASN002 OR Ruxolitinib OR INCB018424 OR INCA24 OR Tofacitinib OR Tasocitinib OR "Tofacitinib citrate" OR Xeljanz OR CP690550 OR Upadacitinib OR "ABT-494" OR RINVOQ OR Cerdulatinib OR "RVT-502" OR PRT062070 OR Peficitinib OR ASP015K OR Filgotinib OR GLPG0634 OR Solcitinib OR "1206163-45-2" OR GLPG0778 OR GSK2586184 OR SHR0302 OR "ATI-502" OR "ATI 50002" OR "A 301" OR "PF-06700841" OR "RVT-501" OR Decernotinib OR "VX-509" OR Pacritinib OR SB1518 OR Oclacitinib OR Apoquel OR "PF-03394197" OR Fedratinib OR TG101348 OR SAR302503 OR "Fedratinib hydrochloride" OR Inrebic).mp	
4	exp "Janus kinase inhibitor"/	
5	(1 OR 2) AND (3 OR 4)	520

c. Search strategy in Cochrane Central Register of Controlled Trials (CENTRAL; search date: 2021/01/30)

#	Search syntax	Citations found
1	("atopic dermatitis" OR eczema*):ti,ab,kw	
2	[mh "Dermatitis, Atopic "] OR [mh "Eczema"]	
3	((Janus NEAR/2 kinase* NEAR/2 inhibit*) OR (JAK* NEAR/2 inhibit*) OR Abrocitinib OR "PF-04965842" OR Baricitinib OR LY3009104 OR Olumiant OR INCB028050 OR Delgocitinib OR "JTE-052" OR Gusacitinib OR ASN002 OR Ruxolitinib OR INCB018424 OR INCA24 OR Tofacitinib OR Tasocitinib OR "Tofacitinib citrate" OR Xeljanz OR CP690550 OR Upadacitinib OR "ABT-494" OR RINVOQ OR Cerdulatinib OR "RVT-502" OR PRT062070 OR Peficitinib OR ASP015K OR Filgotinib OR GLPG0634 OR Solcitinib OR "1206163-45-2" OR GLPG0778 OR GSK2586184 OR SHR0302 OR "ATI-502" OR "ATI 50002" OR "A 301" OR "PF-06700841" OR "RVT-501" OR Decernotinib OR "VX-509" OR Pacritinib OR SB1518 OR Oclacitinib OR Apoquel OR "PF-03394197" OR Fedratinib OR TG101348 OR SAR302503 OR "Fedratinib hydrochloride" OR Inrebic):ti,ab,kw	
4	[mh "Janus Kinase Inhibitors "]	
5	(#1 OR #2) AND (#3 OR #4)	121

d. Search strategy in Web of Science Core Collection (including Science Citation Index Expanded and Social Sciences Citation Index, 2015–present; search date: 2021/01/30)

#	Search syntax	Citations found
1	TS=("atopic dermatitis" OR eczema*)	
2	TS=((Janus NEAR/2 kinase* NEAR/2 inhibit*) OR (JAK* NEAR/2 inhibit*) OR Abrocitinib OR "PF-04965842" OR Baricitinib OR LY3009104 OR Olumiant OR INCB028050 OR Delgocitinib OR "JTE-052" OR Gusacitinib OR ASN002 OR Ruxolitinib OR INCB018424 OR INCA24 OR Tofacitinib OR Tasocitinib OR "Tofacitinib citrate" OR Xeljanz OR CP690550 OR Upadacitinib OR "ABT-494" OR RINVOQ OR Cerdulatinib OR "RVT-502" OR PRT062070 OR Peficitinib OR ASP015K OR Filgotinib OR GLPG0634 OR Solcitinib OR "1206163-45-2" OR GLPG0778 OR GSK2586184 OR SHR0302 OR "ATI-502" OR "ATI 50002" OR "A 301" OR "PF-06700841" OR "RVT-501" OR Decernotinib OR "VX-509" OR Pacritinib OR SB1518 OR Oclacitinib OR Apoquel OR "PF-03394197" OR Fedratinib OR TG101348 OR SAR302503 OR "Fedratinib hydrochloride" OR Inrebic)	
3	#1 AND #2	262

Figure S2. Summary of Risk of Bias Assessment

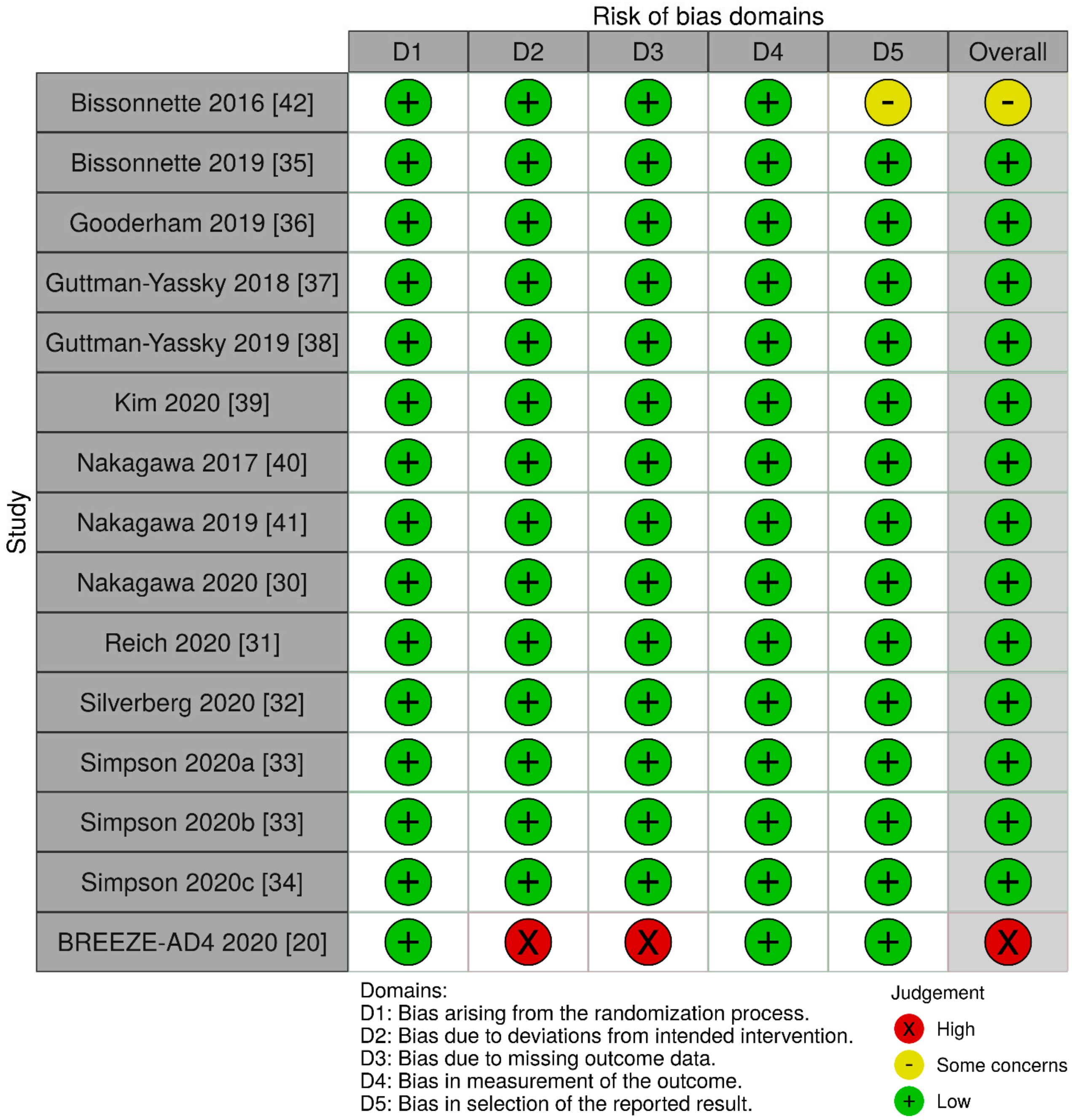

In Bissonnette 2016, protocol amendments regarding the inclusion and exclusion criteria were indicated. Therefore, bias due to selective outcomes of reporting might arise. In BREEZE-AD4 2020, no information about whether the adherence would affect the outcomes and whether the missing outcome data would affect the outcomes. Hence, this trial was judged as “high” bias risk.

Table S2. Subgroup analyses and meta-regressions of efficacy outcomes

		EASI-75 response			Meta-regression	
**Subgroups**	**No. of studies**	**Pooled RR (95%CI)**	***p*-value**	**I^2 (%)**	**τ^2**	***P*-value**
Overall	12	2.84 (2.20 to 3.67)**	< 0.001	38.9		
Severity of atopic dermatitis					0.071	.33
Mild to moderate	1	5.15 (1.69 to 15.69)**	.004	NA		
Moderate to severe	11	2.77 (2.13 to 3.59)**	< 0.001	39.8		
Age of participants					0	.001
Adults only	7	2.21 (1.78 to 2.74)**	< 0.001	0.9		
Contain children or adolescents	5	4.68 (3.23 to 6.79)**	< 0.001	0.0		
Mechanism of action					0.084	.83
Selective for JAK 1 inhibition	4	3.88 (2.62 to 5.76)**	< 0.001	19.8		
Selective for JAK 1/JAK2 inhibition	4	2.14 (1.69 to 2.71)**	< 0.001	0.0		
Pan-JAK inhibition	4	3.63 (1.98 to 6.68)**	< 0.001	7.9		
		**IGA response**			**Meta-regression**	
**Subgroups**	**No. of studies**	**Pooled RR (95%CI)**	***p*-value**	**I^2 (%)**	**τ^2**	***P*-value**
Overall	11	2.99 (2.26 to 3.95)**	< 0.001	0.0		
Severity of atopic dermatitis					0	.21
Mild to moderate	3	4.27 (2.29 to 7.95)**	< 0.001	0.0		
Moderate to severe	8	2.73 (1.99 to 3.73)**	< 0.001	0.0		
Age of participants					0	.16
Adults only	6	2.65 (1.83 to 3.83)**	< 0.001	7.1		
Contain children or adolescents	5	3.94 (2.44 to 6.35)**	< 0.001	0.0		
Mechanism of action					0	.77
Selective for JAK 1 inhibition	3	3.97 (2.45 to 6.42)**	< 0.001	0.0		
Selective for JAK 1/JAK2 inhibition	3	2.19 (1.47 to 3.26)**	< 0.001	0.0		
Selective for JAK 1/JAK3 inhibition	1	5.59 (2.16 to 14.46)**	< 0.001	NA		
Pan-JAK inhibition	4	3.09 (1.19 to 8.06)*	.02	0.0		
		**Pruritus-NRS response**			**Meta-regression**	
**Subgroups**	**No. of studies**	**Pooled RR (95%CI)**	***p*-value**	**I^2 (%)**	**τ^2**	***P*-value**
Overall	8	2.52 (1.90 to 3.35)**	< 0.001	39.4		
Severity of atopic dermatitis					0.074	.57
Mild to moderate	1	3.36 (1.43 to 7.92)**	.006	NA		
Moderate to severe	7	2.47 (1.82 to 3.36)**	< 0.001	44.9		
Age of participants					0.019	.07
Adults only	6	2.18 (1.62 to 2.92)**	< 0.001	25.1		
Contain children or adolescents	2	3.56 (2.32 to 5.48)**	< 0.001	0.0		
Mechanism of action					0.077	.41
Selective for JAK 1 inhibition	4	3.08 (1.73 to 5.48)**	< 0.001	64.5		
Selective for JAK 1/JAK2 inhibition	4	2.17 (1.64 to 2.87)**	< 0.001	0		

*$p < 0.05$; **$p < 0.01$

Table S3. Subgroup analyses of efficacy outcomes for various Janus kinase inhibitors at different time points

Subgroups	No. of studies	Pooled RR (95%CI)	*p*-value	I^2 (%)
**EASI-75 response**				
**Overall**	12	2.84 (2.20 to 3.67)**	< 0.001	38.9
Week 4				
Delgocitinib	3	4.69 (2.46 to 8.92)**	< 0.001	0.0
Gusacitinib	1	**1.17 (0.29 to 4.63)**	.83	NA
Week 12				
Abrocitinib	3	3.69 (2.26 to 6.01)**	< 0.001	40.3
Week 16				
Baricitinib	4	2.14 (1.69 to 2.71)**	< 0.001	0.0
Upadacitinib	1	5.13 (1.99 to 13.21)**	.001	NA
**IGA response**				
**Overall**	11	2.99 (2.26 to 3.95)**	< 0.001	0.0
Week 4				
Delgocitinib	3	3.90 (1.32 to 11.53)*	.01	0.0
Gusacitinib	1	**1.33 (0.17 to 10.43)**	.78	NA
Tofacitinib	1	5.59 (2.16 to 14.46)**	< 0.001	NA
Week 8				
Ruxolitinib	1	3.11 (1.32 to 7.34)*	.01	NA
Week 12				
Abrocitinib	3	3.97 (2.45 to 6.42)**	< 0.001	0.0
Week 16				
Baricitinib	2	1.98 (1.26 to 3.11)**	.003	0.0
**Pruritus-NRS response**				
**Overall**	8	2.52 (1.90 to 3.35)**	< 0.001	39.4
Week 8				
Ruxolitinib	1	3.36 (1.43 to 7.92)**	.006	NA
Week 12				
Abrocitinib	3	2.70 (1.51 to 4.82)**	.001	67.3
Week 16				
Baricitinib	3	2.06 (1.52 to 2.77)**	< 0.001	0.0
Upadacitinib	1	7.65 (1.94 to 30.11)**	.004	NA

*$p < 0.05$; **$p < 0.01$

Table S4. Sensitivity analysis of overall effects of each outcome before and after modified Hartung-Knapp-Sidik-Jonkman (HKSJ) adjustment

Subgroups	No. of studies	Pooled RR (95%CI)	*p*-value	I^2 (%)
**EASI-75 response**				
Before adjustment	12	2.84 (2.20 to 3.67)**	< 0.001	38.9
After adjustment	12	2.84 (2.13 to 3.80)**	< 0.001	38.9
**IGA response**				
Before adjustment	11	2.99 (2.26 to 3.95)**	< 0.001	0.0
After adjustment	11	2.99 (2.17 to 4.11)**	< 0.001	0.0
**Pruritus-NRS response**				
Before adjustment	8	2.52 (1.90 to 3.35)**	< 0.001	39.4
After adjustment	8	2.52 (1.78 to 3.58)**	< 0.001	39.4
**TEAEs**				
Before adjustment	12	1.14 (1.02 to 1.28)*	.02	52.0
After adjustment	12	1.14 (1.00 to 1.31)*	.04	52.0
**AEs leading to drug discontinuation**				
Before adjustment	14	0.89 (0.57 to 1.38)	.62	0.0
After adjustment	14	0.89 (0.54 to 1.45)	.60	0.0

*$p < 0.05$; **$p < 0.01$

Figure S3. Funnel Plot of Pruritus-NRS Response

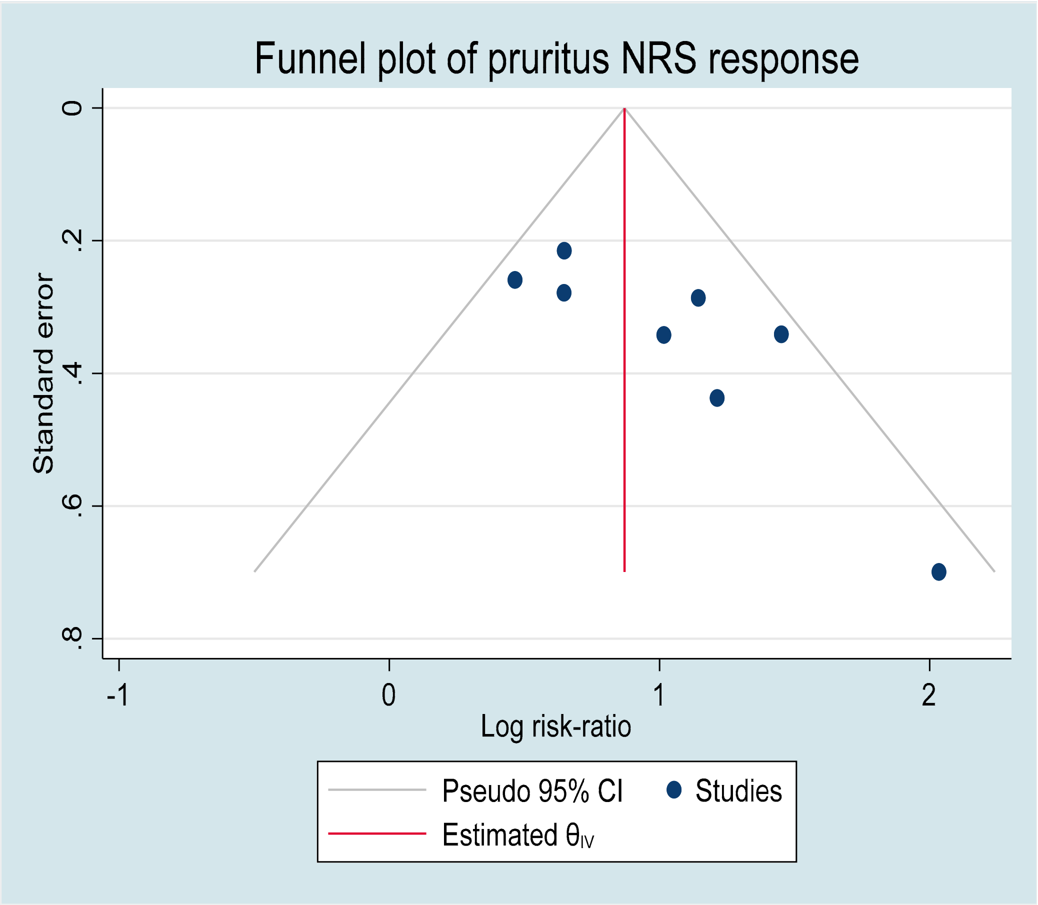

Supplement: Supplementary file 1 [file jpm-11-00279-s001.pdf]
